# Pharmacokinetic Evaluation of Empagliflozin in Healthy Egyptian Volunteers Using LC-MS/MS and Comparison with Other Ethnic Populations

**DOI:** 10.1038/s41598-017-02895-7

**Published:** 2017-05-31

**Authors:** Bassam M. Ayoub, Shereen Mowaka, Eman S. Elzanfaly, Nermeen Ashoush, Mohamed M. Elmazar, Shaker A. Mousa

**Affiliations:** 1 Pharmaceutical Chemistry Department, Faculty of Pharmacy, The British University in Egypt, El-Sherouk city, Cairo, Egypt; 2 The Center for Drug Research and Development (CDRD), Faculty of Pharmacy, The British University in Egypt, El-Sherouk city, Cairo, Egypt; 3 Analytical Chemistry Department, Faculty of Pharmacy, Helwan University, Ein Helwan, Cairo, Egypt; 4 Analytical Chemistry Department, Faculty of Pharmacy, Cairo University, Kasr El-Aini St., Cairo, Egypt; 5 The Center of Applied Research and Advanced Studies (CARAS), Faculty of Pharmacy, Cairo University, Kasr El-Aini St., Cairo, Egypt; 6 Clinical Pharmacy and Pharmacy Practice Department, Faculty of Pharmacy, The British University in Egypt, El-Sherouk city, Cairo, Egypt; 7 Pharmacology Department, Faculty of Pharmacy, The British University in Egypt, El-Sherouk city, Cairo, Egypt; 8 The Pharmaceutical Research Institute, Albany College of Pharmacy and Health Sciences, Rensselaer, NY United States

## Abstract

The present study considered the pharmacokinetic evaluation of empagliflozin after administration to Egyptian volunteers, and the results were compared with other ethnic populations. The FDA recognizes that standard methods of defining racial subgroups are necessary to compare results across pharmacokinetic studies and to assess potential subgroup differences. The design of the study was as an open labeled, randomized, one treatment, one period, single dose pharmacokinetic study. The main pharmacokinetic parameters estimated were C_max_, T_max_, t_1/2_, elimination rate constant, AUC_0-t_ and AUC_0-inf_. The insignificant difference in pharmacokinetic parameters between Egyptians and white German subjects suggests that no dose adjustment should be considered with administration of 25 mg empagliflozin to Egyptian population. A new LC-MS/MS method was developed and validated, allowing sensitive estimation of empagliflozin (25–600 ng mL^−1^) in human plasma using dapagliflozin as an internal standard (IS). The method was applied successfully on the underlying pharmacokinetic study with enhanced sample preparation that involved liquid-liquid extraction. Multiple Reaction Monitoring (MRM) of the transition pairs of m/z 449.01 to 371.21 for empagliflozin and m/z 407.00 to 328.81 for dapagliflozin (IS) was employed utilizing negative mode Electro Spray Ionization (ESI). The validated LC-MS/MS method is suitable for further toxicodynamic and bioequivalence studies.

## Introduction

**Figure 1:**
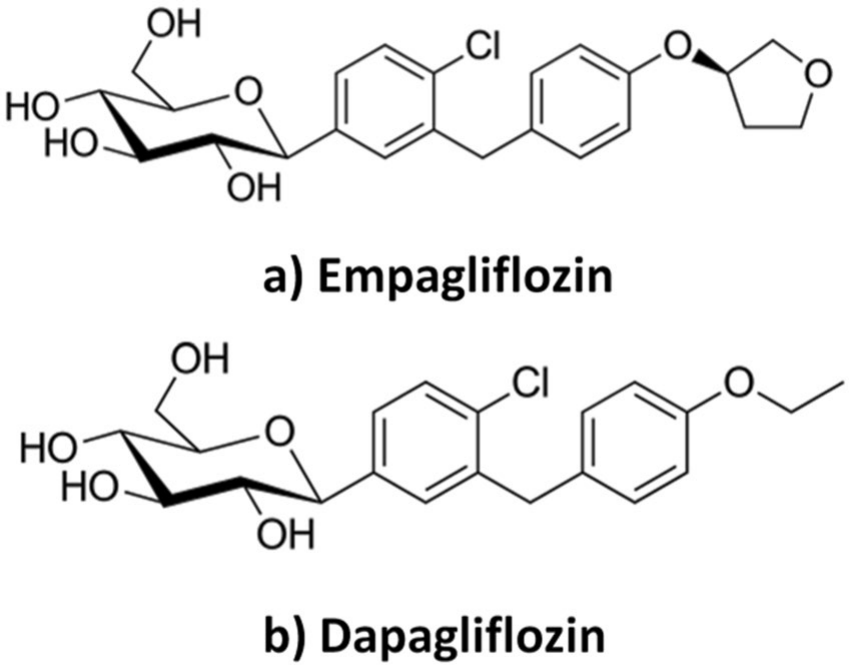
Chemical structures of empagliflozin (**a**) and the internal standard, dapagliflozin (**b**).

The Food and Drug Administration (FDA) defined ethnic factors as those related to races or large populations grouped according to the International Conference on Harmonization (ICH) guidelines^1^. Some drugs could be “ethnically sensitive” according to their metabolic pathways or steep dose-response curves^2^. The kidney has a role in the regulation of blood glucose levels and can therefore serve as a target for new anti-diabetic drugs. Empagliflozin (EG) and dapagliflozin (DG), (Fig. 1), are inhibitors of sodium glucose co-transporter-2 (SGLT-2) that inhibit glucose re-absorption into the blood^3, 4^. SGLT-2 is expressed in the kidneys and plays an important role of renal glucose re-absorption. EG and DG can selectively inhibit SGLT-2 and therefore enhance urinary glucose excretion. The amount of glucose removed by the kidney through this glucuretic mechanism is dependent upon the blood glucose concentration and glomerular filtration rate (GFR)^3, 4^.

The pharmacokinetic evaluation of EG after administration to Egyptian volunteers and its comparison to the previously developed studies on different races will minimize the duplication of clinical data. A fully validated bioanalytical method is a prerequisite to perform a successful pharmacokinetic study. In the present work, a new fast LC-MS/MS method was developed for sensitive estimation of EG using DG as an internal standard (IS) to enable further pharmacokinetic and pharmacodynamic evaluation to facilitate satisfactory clinical outcomes.

LC-MS/MS parameters and analytical procedure details were not described in the pharmacokinetic studies reported for EG^5–23^. Chromatograms and parameters of the analytical assay such as chromatographic conditions, matrix effects, extraction recovery, and stability are not fully described for duplication in most clinical studies^5–23^. Therefore, the novelty of the present work was achieved by providing the full details regarding the development and validation of the proposed analytical procedure for the simultaneous extraction and LC-MS/MS determination of EG and DG (IS). Furthermore, in the present work, the first pharmacokinetic study on healthy Egyptian volunteers, after administration of 25 mg EG (JARDIANCE), was applicable using the proposed bioanalytical method.

Investigation of the relationship between drug dosage and the concentration time profiles will be useful for the design of subsequent clinical trials, appropriate analysis in post-marketing pharmacovigilance, determination of the appropriate use of medicines according to genotype of drug-metabolizing enzymes, and providing information for therapeutic drug monitoring (TDM). The developed LC-MS/MS method measures the plasma concentration of the parent compound (EG) because no major metabolites of EG were detected in human plasma as glucuronidation is the major metabolic pathway^14^.

## Materials and Methods

### Instrumentation

WATERS ACQUITY UPLC system (S/N F08UPH, USA), TQ detector (S/N QBA530, USA) accompanied with ESI source and WATERS ACQUITY UPLC BEH Shield RP C_18_ column (S/N 01563430116023, Ireland) with dimensions (150 mm × 2.1 mm, 1.7 µm) were used. MASS LYNX software version 4.1 was used. Vacuum evaporator CHRIST (S/N 20534, Germany), vacuum pump VACWBRAND (DVP2C-TYR012, Germany), Vortex VELP SCIENTIFICA (S/N 265349, Europe), −80 °C freezer THERMO SCIENTIFIC (S/N 836003-375, USA), and Centrifuge HETTICH (S/N 012444807, Germany) were used. Validated Excel software was used to calculate the pharmacokinetic parameters.

### Chemicals, reagents, stock solutions and working solutions

Pharmaceutical grade EG certified to contain 99.90%, JARDIANCE tablets nominally containing 25 mg of EG per tablet, was supplied from Boehringer Ingelheim pharmaceutical company (Germany). Pharmaceutical grade DG certified to contain 99.80% was kindly donated by researcher Moataz Hendy, research assistant at the Center for Drug Research and Development funded by the British University in Egypt (CDRD, BUE). Human plasma was donated from Vacsera (Egypt). Ammonium acetate, *tert*-butyl methyl ether (TBME), formic acid, deionized water, and HPLC grade acetonitrile were purchased from Sigma Aldrich (USA). Stock solutions of pharmaceutical grade EG (1 mg/mL) and DG (1 mg/mL) were prepared separately in acetonitrile. Working solutions of EG (50 µg/mL) and DG (1 µg/mL) were prepared separately in acetonitrile with appropriate dilutions from stock solutions. All solutions were stored at 4 °C.

### Chromatographic and mass spectrometric conditions

A mixture of deionized water and acetonitrile in the ratio of (10:90, *v/v*) was used as the mobile phase. The column temperature was kept at 25 °C, the injection volume used was 10 µL, and the flow rate was 0.3 mL/min with 1.5 min as the run time. Cone voltage was set at 40 V; source temperature was set at 150 °C, and the collision energy was set at 30 eV for both drugs to enable multiple reaction monitoring (MRM) of the transition pairs of m/z 449.01 to 371.21 for EG and m/z 407.00 to 328.81 for DG (IS) in the negative mode utilizing Electro Spray Ionization (ESI). The following parameters were applied: turbo ions spray at 400 °C, capillary temperature at 275 °C, sheath and auxiliary gas at 15 and 2 psi, respectively, ion spray voltage of 3800 V, capillary voltage of 4 KV, capillary offset of 35 and de-solvating line temperature at 400 °C.

### Procedures and method validation

#### Sample preparation, calibrators (linearity,) and QC samples (accuracy and precision)

Each EG calibrator and quality control (QC) plasma sample (980 µL) was spiked with 20 µL containing the appropriate amount of EG in acetonitrile prepared with dilution of EG working solution. All samples including the volunteers’ plasma samples (1 mL plasma) were spiked with 100 µL of IS that contained 100 ng of DG followed by vortexing for 20 sec. Five milliliters of TBME were added, vortexed for 1 min, and centrifuged for 30 min at 6000 rpm. Four milliliters of the organic layer were vacuum evaporated until dryness at 60 °C. Three hundred microliters of acetonitrile were added to reconstitute the resulting residue and vortexed for 3 min. Ten microliters of the reconstituted solution were injected into the LC-MS/MS system.

Different EG concentrations equivalent to (25, 100, 200, 300, 400, 600 ng) per 20 µL were prepared in acetonitrile using appropriate volumes of working solutions. Plasma samples were prepared by spiking 980 µL human plasma with 20 µL of EG and finally spiked with 100 µL of IS working solution in acetonitrile (1 µg/mL). After the sample preparation, the peak area ratios of EG to IS against the corresponding concentrations of 25–600 ng/mL for EG were used to generate the calibration curve. Both plasma standards and QC samples were kept at −80 °C until used.

Different EG concentrations in acetonitrile were used to prepare different QC levels: lower as 50 ng/mL (LQC), middle as 250 ng/mL (MQC), and high as 500 ng/mL (HQC). Then the procedure was repeated to check the accuracy of the results along with lower limit of quantification (LLOQ). Repeatability was assessed with the analysis of replicates of QC samples and LLOQ on the same day (n = 6), while the intermediate precision was assessed with their analysis on three successive days.

#### Selectivity, matrix factor, and recovery

Selectivity of the method was assessed by analyzing 6 different blank plasma samples obtained from different sources. The matrix factor was determined by measuring the peak areas of EG from the post-extracted LQC and HQC samples and its comparison to the peak areas of neat samples at the same concentrations in acetonitrile to estimate the effect of the biological matrix on the ionization of EG. The extraction recovery was determined by measuring the peak areas of EG from LQC and HQC samples extracted from human plasma followed by its comparison to the peak areas of the same QC samples prepared by spiking the supernatant of the extracted blank plasma.

#### Carry-over and stability experiments

Carry-over effect was assessed by injecting blank samples after HQC to ensure that its response was less than 20% of the LLOQ. Aliquots of LQC and HQC samples were kept for a period of 6 hrs at room temperature to check short-term stability. The post-operative stability of the processed samples was examined by keeping LQC and HQC samples in the auto sampler at 25 °C for 24 hrs. Long-term stability was determined by storing aliquots of LQC and HQC samples at −80 °C for 1 week. The stability of the analytes was determined after freeze and thaw cycles, using aliquots of LQC and HQC samples stored at −80 °C for 24 hrs and thawed unassisted at room temperature. Evaluation of stability was carried out by comparing the mean recovery of EG and IS obtained from stored samples with the mean values obtained using freshly prepared samples at the same concentration levels; the concentration change should be less than 15% of the nominal concentration^24^.

#### Human subjects and pharmacokinetic study of EG

The pharmacokinetic parameters of EG were studied in healthy human subjects according to the ethical regulations of World Medical Association Declaration of Helsinki (October 1996) and the International Conference of Harmonization Tripartite Guideline for Good Clinical Practice. Written informed consent was provided by each volunteer before enrollment. Approval of the study by the ethical committee was mandatory according to the Egyptian ministry of Health and The British University in Egypt research ethics guidelines. The experimental protocols were approved by the ethics committee of the Faculty of Pharmacy, The British University in Egypt. The clinical trial protocol was registered in a publically accessible primary register that participates in the WHO International Clinical Trial Registry Platform (ClinicalTrials.gov, 16/02/2017, ID: NCT03059056). Good health of the human subjects was confirmed with a complete medical history and physical examination. Fasting of all volunteers eliminated the possible interaction from high fat meals. The evaluation of safety of the study was based on monitoring of blood glucose level, vital signs, pulse rate, monitoring of adverse events, and physical examination. Samples from 6 healthy, adult, male, Egyptian volunteers (age: 22–33 years, average weight: 77.8 kg, average body mass index (BMI): 29.2) were collected at 0, 0.5, 1, 1.5, 2, 3, 4, 8 and 12 hrs, transferred to heparinized centrifuge tubes and analyzed with the proposed method after single oral dose administration of one JARDIANCE tablet nominally containing 25 mg EG. Blood samples (3 mL of each sample) were centrifuged at 3000 rpm for 5 min, 1 mL of the plasma was separated and spiked with 100 µL (equivalent to 100 ng) of IS working solution, and then the procedure discussed under (*Sample preparation*) was applied. The main pharmacokinetic parameters of the study, C_max_, T_max_, t_1/2_, elimination rate constant, AUC_0-t_ and AUC_0-inf_, were estimated using validated Excel software. Blood glucose level was determined for all volunteers at 0 and 1.5 hrs to monitor any hypoglycemic effect. The study was conducted as per FDA guidelines.

## Results and Discussion

### Optimization of sample preparation, chromatographic conditions, and mass spectrometric parameters

The LC-ESI-MS/MS method was developed for accurate and sensitive estimation of EG in human plasma. For the extraction procedure, liquid-liquid extraction was tried using ethyl acetate, dichloromethane, and diethyl ether, and the best results were obtained with TBME. This may be attributed to the ability of EG and DG (IS) to migrate to TBME according to their partition coefficients and log P values. Using DG as IS for EG bioanalysis enhanced the validation results because of their structural similarity (Fig. 1), closely related plasma extraction recoveries, and similar matrix factors (MF). After vortex and centrifugation, vacuum evaporation of the TBME layer until dryness at 60 °C followed by reconstitution with 300 µL acetonitrile was employed as a sample enrichment technique, enabling determination of EG at the LLOQ, equal to 25 ng/mL. Another advantage of using liquid extraction is sample clean-up, decreasing the matrix effect on the detector response. Furthermore, using acetonitrile as solvent for IS decreased the formation of irregular emulsion between aqueous/organic interfaces and modulated the polarity of the extraction solvents to achieve the desired recovery^25^. In addition, TBME was reported by Kobuchi *et al*. for sample preparation of some SGLT-2 inhibitors^26–29^ with structural similarity to EG, namely, canagliflozin^26^, tofogliflozin^27^, ipragliflozin^28^, and luseogliflozin^29^ using EG^26–28^ or DG^29^ as IS.

**Figure 2:**
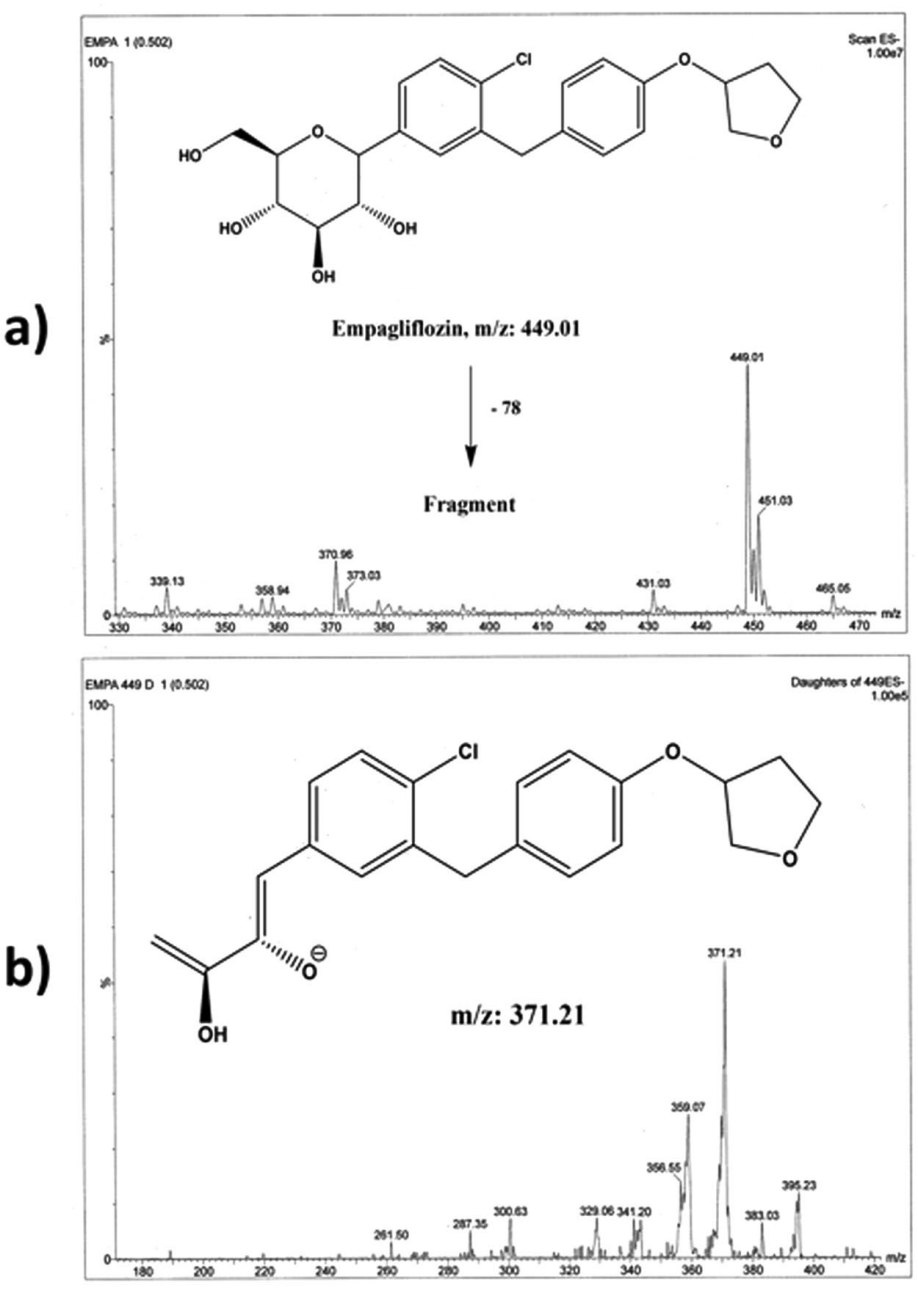
Full scan mass spectrum (**a**) and daughter ion mass spectrum (**b**) of empagliflozin in negative ESI ion detection mode with the proposed fragment.

**Figure 3:**
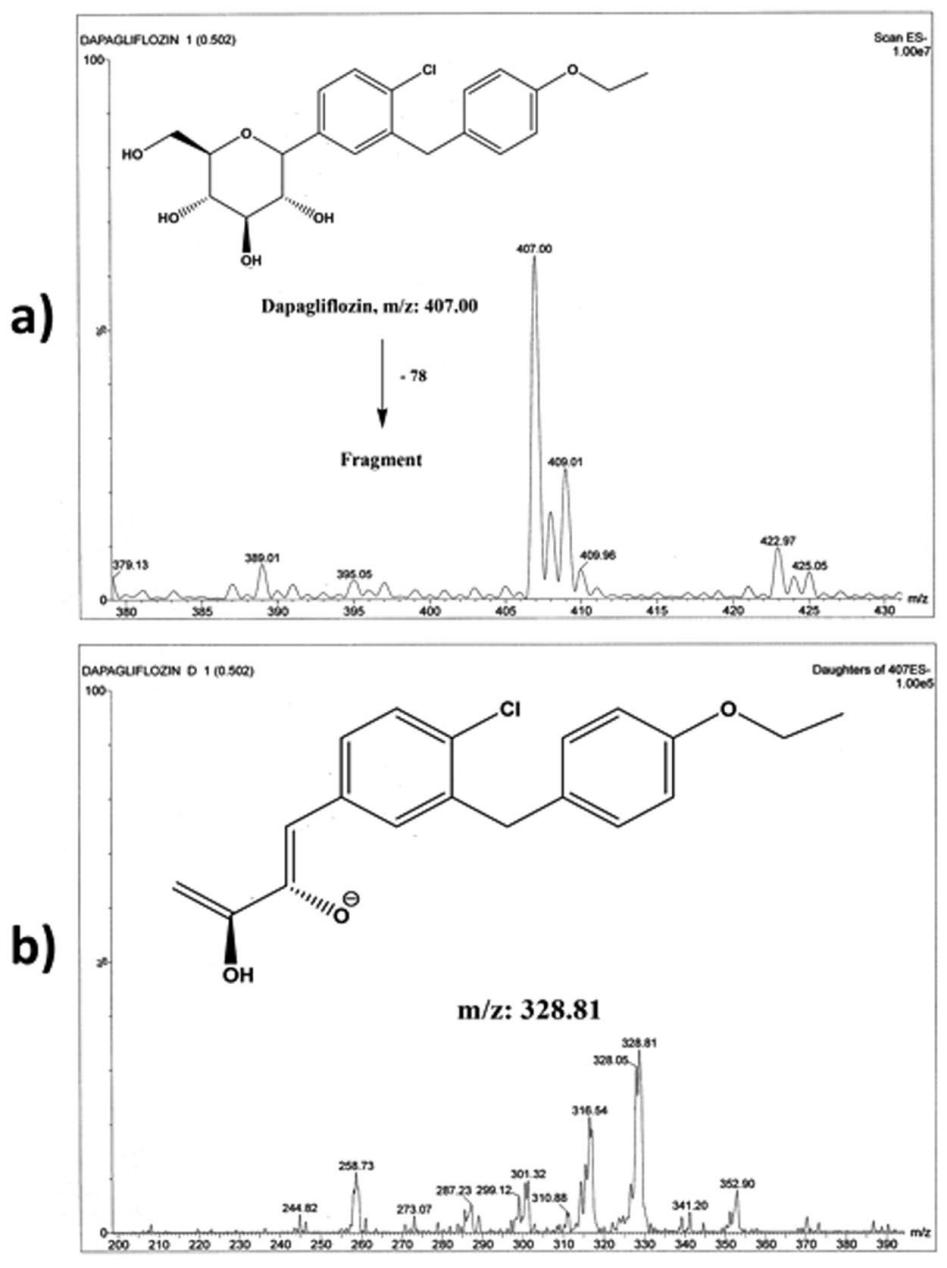
Full scan mass spectrum (**a**) and daughter ion mass spectrum (**b**) of dapagliflozin in negative ESI ion detection mode with the proposed fragment.

For optimum detection of EG and the IS, both the chromatographic conditions and the mass detector parameters were adjusted. Both positive and negative ionization modes and various mobile phases (containing ammonium acetate or formate) were initially assessed. Although LC-MS/MS in the positive mode ESI has been reported in literature while using EG as IS^26–28^, the best intensities for precursor and product ions were attained in the negative mode for EG and the IS (Figs 2 and 3); this may be attributed to the reported adduct formation in the case of using positive mode with EG or DG^29, 30^. Also a study published by Iqbal *et al*.^31^ recommended the use of negative mode over the positive mode for superior sensitivity advantages for canagliflozin.

Molecular ions of 449.01 and 407.00 were observed for EG and DG, respectively, on the full scan mass spectra (Figs 2 and 3). The optimized collision energy produced significant fragments. The MS/MS transition of 449.01 → 371.21 and 407.00 → 328.81 for EG and the IS, respectively, were selected. The selected DG transition is consistent with previous LC-MS/MS methods in the negative mode^29, 30^, while the transition of EG is reported here for the first time using negative mode. Both EG and DG fragments can be explained as shown in Figs 2 and 3.

**Figure 4:**
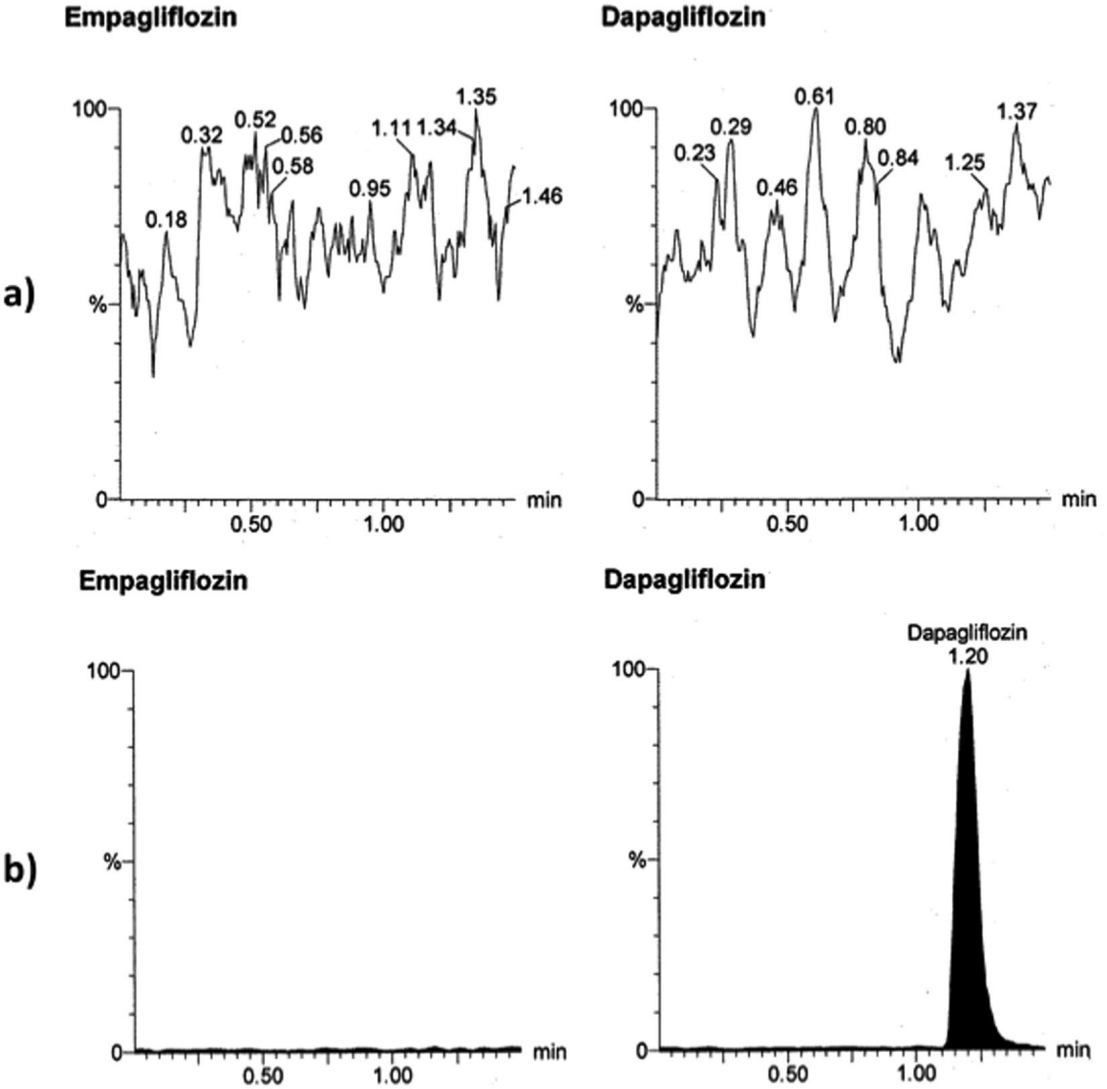
Multiple reaction monitoring (MRM) chromatogram of empagliflozin (m/z = 449.01 to 371.21) and dapagliflozin (internal standard, m/z = 407.00 to 328.81): (**a**) blank plasma; (**b**) zero plasma spiked with internal standard.

**Figure 5:**
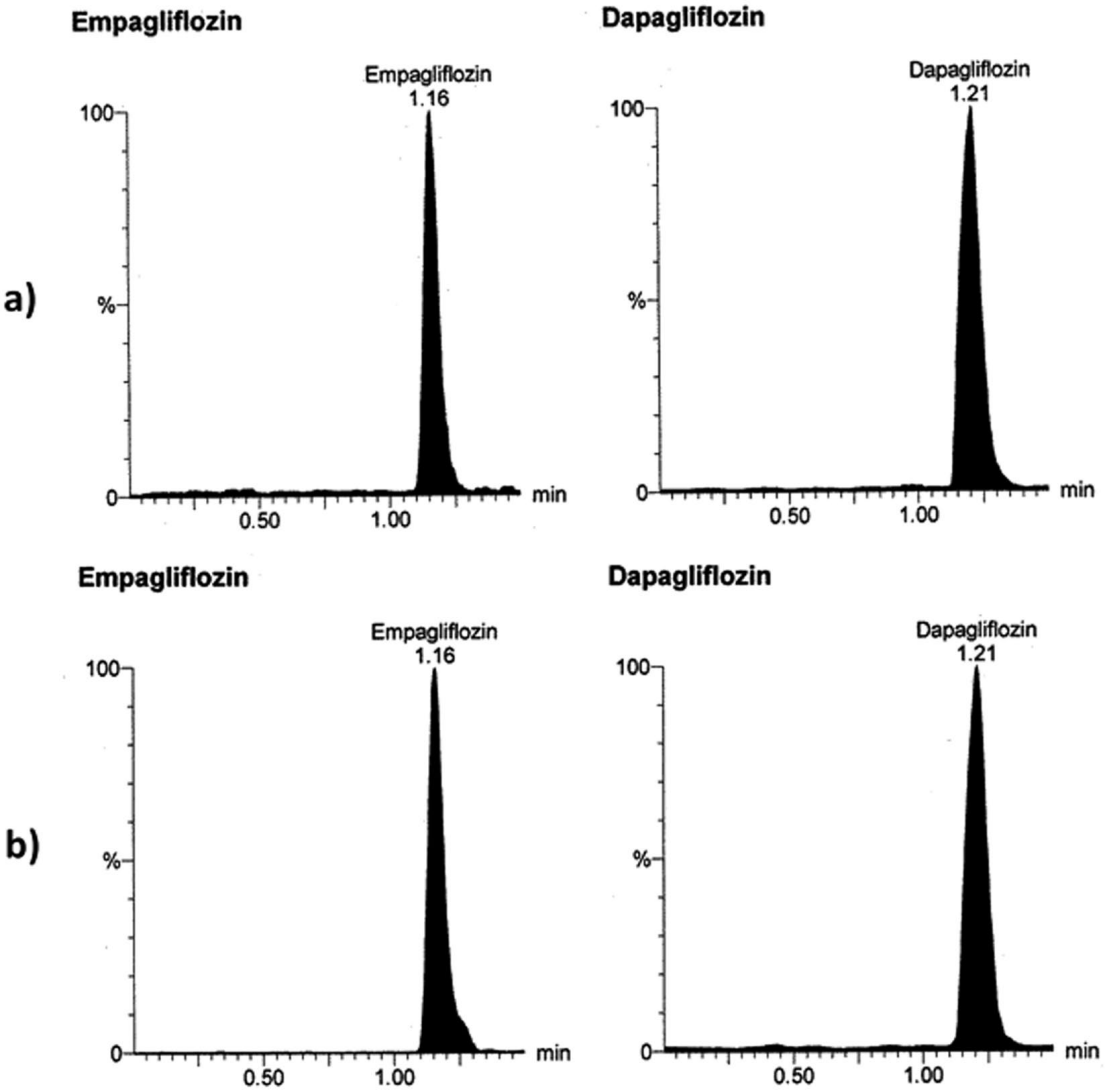
Multiple reaction monitoring (MRM) chromatogram of empagliflozin (m/z = 449.01 to 371.21) and dapagliflozin (internal standard, m/z = 407.00 to 328.81): (**a**) lower limit of quantitation (LLOQ); (**b**) human plasma sample obtained 1.5 hrs after oral administration of one JARDIANCE tablet nominally containing 25 mg of empagliflozin.

To attain the optimum chromatographic conditions, various combinations of organic solvents and different concentrations of formic acid solution were tried for the positive mode trials, while different concentrations of ammonium acetate buffer and different acetonitrile/water ratios were tried for the negative mode. The final mobile phase was selected based on the high response and best peak shape of the analytes in a reasonable run time. Because DG (IS) readily forms adducts in the presence of formic acid, the mobile phases were simple mixtures of water and acetonitrile, which is consistent with previous reports for LC-MS/MS determination of DG^30^. Optimum results with well-defined peaks and high sensitivity (Figs 4 and 5) were obtained using a mixture of water and acetonitrile in the ratio of (10:90, *v/v*) as a mobile phase, keeping column temperature at 25 °C, using 10 µL as the injection volume and 0.3 ml/min as the flow rate with 1.5 min as run time. LC-MS/MS was selected for the underlying investigation because it is a well-known, sensitive technique that has been commonly used for many pharmacokinetic studies^32–37^.

### LC-MS/MS method validation

#### Linearity, accuracy, and precision

The plasma calibration curves were constructed by plotting the (drug/IS) peak area ratios of 6 samples against EG concentrations covering the expected range including LLOQ (FDA, 2001)^24^. The regression equation was found to be (peak area ratio = 0.0106 concentration + 0.0634) with correlation coefficient (r) = 0.9997. The lowest concentration at S/N ratios of 10 with the %RSD <20% was taken as LLOQ (25 ng/mL). The results were in agreement with FDA recommendations^24^ that the correlation coefficient (r) of a calibration curve should be less than 0.99, and the deviation of the back-calculated concentrations at each point was found to be within ±15% and within ±20% for the LLOQ.

Accuracy was measured by repeated analysis of each drug in human plasma. LLOQ and three concentration levels were studied as low, medium, and high QC (FDA, 2001)^24^. The mean value was within 15% of the actual values for LLOQ and the QC samples ranged from 98.46% to 101.19%, showing that the bias% ranged from −1.54 to 0.19, confirming the accuracy of the proposed method. The precision, percent coefficient of variation (% C.V.), was within 15% of the actual values. The intra-day and inter-day precision values confirmed that the proposed methods are precise, with %RSD ranging from 4.71% to 6.99%.

#### Selectivity, matrix factor, and recovery

Selectivity and lack of interference from plasma components was confirmed by comparison between blank plasma and spiked plasma chromatograms at LLOQ of both EG and IS. Matrix factor (MF) describes the analyte ionization efficiency in the ion source due to co-eluting matrix components. MF for EG ranged from 0.90 (HQC) to 0.88 (LQC), indicating no significant matrix effect over the ionization of EG. Recovery describes the efficiency of separating the drug from the sample. Recovery experiments were performed by comparing the peak area of the low and high QC samples extracted from human plasma with those spiked in the supernatant of the extracted blank plasma at the same concentration levels. The average recoveries of EG were 77.19% for the LQC and 83.84% for HQC samples, which satisfy the FDA recommendation of being above 70%.

#### Carry-over and stability experiments

Carry-over effect was addressed during method development by injecting blank samples after HQC sample, i.e. 500 ng/mL of EG and checking the response of EG (peak area). Carry-over in the blank sample following the high concentration standard was <20% of the LLOQ, as recommended by FDA^24^. Stability experiments were performed using 2 concentrations (low and high QC), and the results are acceptable because the concentration change was <15% of the actual values, confirming that the processed samples were stable while studying short-term stability, freeze-thaw stability, post-operative stability, and long-term stability.

### Pharmacokinetic evaluation of EG

Development of correlations between drug concentrations and their pharmacologic responses enable clinicians to apply pharmacokinetic principles to actual patient situations. Pharmacokinetic studies are necessary for the submission of a new drug application (NDA) to the FDA and for re-examination of approved drugs. For extrapolation of clinical data from other countries, ethnic differences in pharmacokinetics must be discussed.

**Figure 6:**
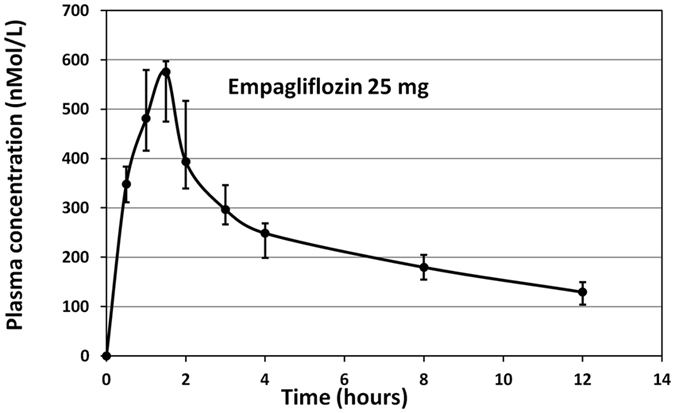
The mean plasma concentration-time curves of empagliflozin after oral administration of one JARDIANCE tablet nominally containing 25 mg of empagliflozin. Each symbol with a bar represents the mean ± S.D. of 6 subjects.

The proposed method was applied to a pharmacokinetic study and the mean plasma concentration (nMol/L) was plotted against time (Fig. 6). The main pharmacokinetic parameters of the study are presented in Table 1. C_max_ and T_max_ values suggest that EG is rapidly absorbed from the gastrointestinal tract into the circulation.

**Table 1:** Pharmacokinetic parameters of empagliflozin (EG) following oral administration of one JARDIANCE tablet nominally containing 25 mg of EG.

Pharmacokinetic parameters	Empagliflozin
C_max_ (nMol/L), Mean ± S.D. (% C.V.)	576 ± 86 (14.93)
T_max_ (hours), Median (range)	1.5, (1–2)
t _1/2_ (hours), Mean ± S.D. (% C.V.)	6.1 ± 1.2 (19.67)
Elimination rate constant (h^−1^), Mean ± S.D. (% C.V.)	0.10012 ± 0.02156 (19.25)
AUC_0−t (12)_ (nMol.h/L), Mean ± S.D. (% C.V.)	2806 ± 234 (8.34)
AUC_0-inf_ (nMol.h/L), Mean ± S.D. (% C.V.)	4103 ± 427 (10.41)

Abbreviations: AUC = area under the curve; % C.V. = percent coefficient of variation; S.D. = standard deviation.

No clinically meaningful interactions were observed when EG was co-administered with other commonly used medicinal products and no dose adjustment was recommended^5–23^. The insulin-independent mechanism of action of EG contributes to a low risk of hypoglycemia that was proved by monitoring of blood glucose level of all volunteers while carrying out the study, and the results were in the normal range. The glucosuria observed with EG was accompanied by mild diuresis.

The previous pharmacokinetic studies confirmed the absence of pharmacokinetic interaction of EG with pioglitazone^8^, hydrochlorothiazide^10^, torasemide^10^, gemfibrozil^15^, rifampicin^15^, probenecid^15^, linagliptin^21^, or sitagliptin^22^. In addition, pharmacokinetic parameters were checked in special populations with heart failure^5^, renal impairment^11, 12^, or hepatic impairment^14^ with no need for dose adjustment. Furthermore, efficacy^6^, tolerability^18–20^, single dose and multiple dose kinetics^7, 9^ were reported. The present study compared the pharmacokinetic parameters of Egyptian volunteers to previously reported non-Egyptian populations using 25 mg EG. The calculated pharmacokinetic parameters were closely related to previous studies conducted in white German subjects using 25 mg EG. The insignificant difference in ANOVA statistical results (Table 2) of the pharmacokinetic evaluation in Egyptians and white German subjects^13, 15, 20, 38^ suggests that no dose adjustment should be considered with administration of 25 mg EG to Egyptian population.

**Table 2:** One way ANOVA results at P < 0.05 for C_max_ and AUC _0-inf_ after administration of 25 mg empagliflozin in German and Egyptian subjects.

C_max_ (nMol/L)				AUC_0-inf_ (nMol.h/L)	
*Groups	Number of subjects	Mean	S.D., (% C.V.)	Mean	S.D., (% C.V.)
Group 1^13, 38^	6	505	130, (25.74)	3830	825, (21.54)
Group 2^15^	18	610	98.82, (16.20)	4770	797, (16.7)
Group 3^20^	9	606	147, (24.26)	4310	1040, (24.13)
Group 4^20^	9	630	106, (16.83)	4990	1080, (21.64)
Egyptian subjects	6	576	86, (14.93)	4103	427, (10.41)

*Studied groups from pharmacokinetic studies conducted in white German subjects using 25 mg EG^13, 15, 20, 38^ showed no significant difference at P > 0.05, with P = 0.283 for Cmax and P = 0.064 for AUC_0-inf_.

Abbreviations: AUC = area under the curve; % C.V. = percent coefficient of variation; S.D. = standard deviation.

The insignificant difference between Japanese and Chinese populations (Table 3) may be attributed to the similarity in their BMI as Asian race. A significant difference was observed (Table 4) when comparing all races (white German, Egyptian, Japanese, and Chinese), which may be attributed to the difference in weight and BMI between races that was confirmed in a previous EG population study^17^ that mainly reported the ethnic difference between white and Asian races but did not consider the Egyptian population, which was proved to be similar to the white German volunteers. The reported population study^17^ included different BMI values for the ethnic groups, which was found to be 31.4 kg/m^2^ for the white population and 24.6 kg/m^2^ for the Asian population.

**Table 3:** One way ANOVA results at P < 0.05 for C_max_ and AUC_0-inf_ after administration of 25 mg empagliflozin in Japanese^11^ and Chinese subjects^9^.

C_max_ (nMol/L)				AUC_0-inf_ (nMol.h/L)	
Groups	Number of subjects	Mean	S.D., (% CV)	Mean	S.D., (% CV)
*Group 1^11^	8	1070	193.7, (18.1)	7560	1126.4, (14.9)
*Group 2^9^	9	1130	318.7, (28.2)	7450	1959, (26.3)

*Studied groups from pharmacokinetic studies conducted in Japanese^11^ and Chinese^9^ subjects using 25 mg empagliflozin showed no significant difference at P > 0.05, with P = 0.651 for Cmax and P = 0.891 for AUC_0-inf_.

Abbreviations: AUC = area under the curve; % C.V. = percent coefficient of variation; S.D. = standard deviation.

**Table 4:** One way ANOVA results at P < 0.05 for C_max_ and AUC_0-inf_ after administration of 25 mg empagliflozin in German^13, 15, 20, 38^, Japanese^11^, and Chinese subjects^9^.

Parameter	F	P
C_max_ (nMol/L)	19.614	0
AUC_0-inf_ (nMol.h/L)	15.796	0

F-test is a statistical test in which the test statistic has an F-distribution under the null hypothesis; P is the probability using a given statistical model using ANOVA.

Abbreviations: AUC = area under the curve.

## Conclusion

Pharmacokinetic parameters can vary between different races, and the present analysis was the first study carried out on Egyptian volunteers and compared with the results obtained from other ethnic populations. There is no significant difference was observed between the studied group and the compared ethnic group which suggests that no dose adjustment should be considered with administration of 25 mg EG to Egyptian population. The proposed LC-MS/MS method is simple, fast, accurate, and reproducible for determination of EG in human plasma. The validated method was proved to be suitable for further toxicodynamic evaluation. The method was applied successfully for the pharmacokinetic study under investigation and owing to the short run time used, rapid analysis of many plasma samples per day was achieved.
